# New Forearm Elements Discovered of Holotype Specimen *Australovenator wintonensis* from Winton, Queensland, Australia

**DOI:** 10.1371/journal.pone.0039364

**Published:** 2012-06-27

**Authors:** Matt A. White, Alex G. Cook, Scott A. Hocknull, Trish Sloan, George H. K. Sinapius, David A. Elliott

**Affiliations:** 1 School of Engineering, The University of Newcastle, Callaghan, New South Wales, Australia; 2 Australian Age of Dinosaurs Museum of Natural History, The Jump Up, Winton, Queensland, Australia; 3 Geosciences, Queensland Museum, Hendra, Queensland, Australia; University of Pennsylvania, United States of America

## Abstract

New skeletal elements are reported of the holotype specimen *Australovenator wintonensis*, from the type locality, near Winton, central western Queensland. New elements include left and right humeri, right radius, right radiale, right distal carpal 1, near complete right metacarpal I, left manual phalanx II-1, left manual phalanx II-2, near complete left manual phalanx II-3 and a left manual phalanx III-3. These new elements combined with those previously described are compared against other neovenatorids.

## Introduction

Australian dinosaur discoveries have increased significantly with new specimens being discovered throughout central Queensland’s mid-Cretaceous (latest Albian-Cenomanian) Winton Formation [1]. Excavations carried out by Australian Age of Dinosaurs Museum and the Queensland Museum between 2006 and 2010 yielded two new dinosaur discoveries *Australovenator wintonensis* [1] a neovenatorid theropod [2] and *Diamantinasaurus matildae* a derived lithostrotian titanosaur [1].

Remains of *Australovenator* were found scattered throughout a deposit interpreted as an oxbow lake and were interspersed with the remains of *Diamantinasaurus*. This Australian Age of Dinosaurs Locality (AODL) 85 “Matilda Site” yielded numerous specimens many of which await preparation. We here describe additional forearm elements of the type specimen, Australian Age of Dinosaurs Fossil (AODF) 604. These elements include left and right humeri, right radius, right radiale, right distal carpal 1, near complete right metacarpal I, left manual phalanx II-1, left manual phalanx II-2 and left manual phalanx III-3 and a near complete right manual phalanx II-3. Right manual phalanx I-2 and right manual phalanx III-4 were briefly described [1] however they are here described in greater detail. There was also the suggestion of a badly preserved distal tip belonging to manual phalanx II-3 but was not figured [1]. We now confirm this specimen as a near complete left pedal phalanx I-2. Discovery of additional manus phalanx enabled the reassignment of manual phalanx II-2 from its original description [1] to a right manual phalanx III-1. Additionally we have corrected the description of the right metacarpal II originally described as a left. First and second manus unguals are near equal in size but differ morphologically.

### Stratigraphy and Palaeoenvironment

**Figure 1:**
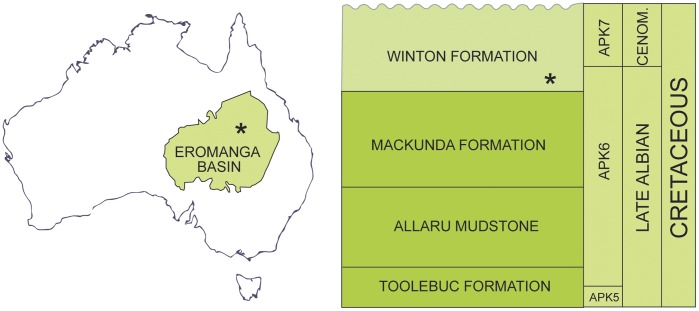
Locality Map and Stratigraphy. Location of Eromanga Basin and mid Cretaceous stratigraphy of the northern part of the Eromanga Basin. *Australovenator wintonensis* locality indicated *.

The holotype of *Australovenator* is derived from the lowermost part of the Winton Formation, in the northern Eromanga Basin (Figure 1). The Eromanga Basin is one of three constituent basins in the Great Artesian Basin system, a Jurassic to Late Cretaceous sedimentary assemblage which dominates the northeast of Australia. The Winton Formation is the youngest stratigraphic unit (Figure 1) in the Eromanga Basin. Its lowermost parts are interpreted as proximal deltaic and near-coastal plain fluvial deposits which developed on a broad, flat plain following final regression of the sea in the latest Albian. An abundant macroflora and microflora [3]–[9] indicates a latest Albian to Cenomanian age for the unit, falling within Australian Palynological Units APK6 (uppermost) and APK7. Specifically the age of the *Australovenator* locality is late Albian [9], in uppermost APK6. The flora indicates open forest to floodplain environments, which is supported by interpretation of local sedimentology. The locality is interpreted to represent an oxbow lake in an abandoned channel of a sluggish riverine system, with adjacent floodplain deposits and overprinted crevasse splay units.

Macrofauna is scarce in the lower Winton Formation, but includes a modest dinosaur fauna-[10], [1]. Other elements include crocodylians, fishes, lungfish, a dolichosaur, turtles, and pterosaur remains. Nearby sites contain dinosaur and other footprints.

## Methods

### Fossil Preparation

The holotype specimen elements were prepared using pneumatic air scribes and chisels. Many of the theropod bones were encased in a concretionary phosphatic crust. The specimens are consolidated with Paraloid B72. ‘Carbowax’ was used to support fragile fossil specimens during preparation, and was used to fill cracks for extra support and absorbed some of the vibration caused by the pneumatic preparation tools.

### Computed Tomography

The specimens were computed tomography (CT) scanned at Queensland Xray, Mackay Mater Hospital, central eastern Queensland using a Philips Brilliance CT 64-slice machine capable of producing 0.9 mm slice. Voyager Viewer and Mimics were used to view all CT scans and take measurements. Mimics version 10.01 software, was used to view internal structures in cross-section. Modelling clay was used to reconstruct missing sections of specimens. Mimics 10.01 was used to create three dimensional meshes of specimens enabling both original and reconstructed specimens to be meshed separately. The meshes were then imported into a graphic design package Rhinoceros 4.0., which was used to develop rendered meshes of fossil specimens enabling morphology to be clearly viewed alongside actual specimens.

### Terminology

For individual postcranial elements the terms ‘dorsal, ventral, medial, lateral, distal, disto-medial, disto-lateral, proximal, cranio-ventral, dorso-distal, dorso-lateral’ are used to identify the region described. When describing an aspect view we use ‘cranial view’ (e.g. distal articular facet) and ‘caudal view’ (e.g. proximal articular facet).

## Results

**Figure 2:**
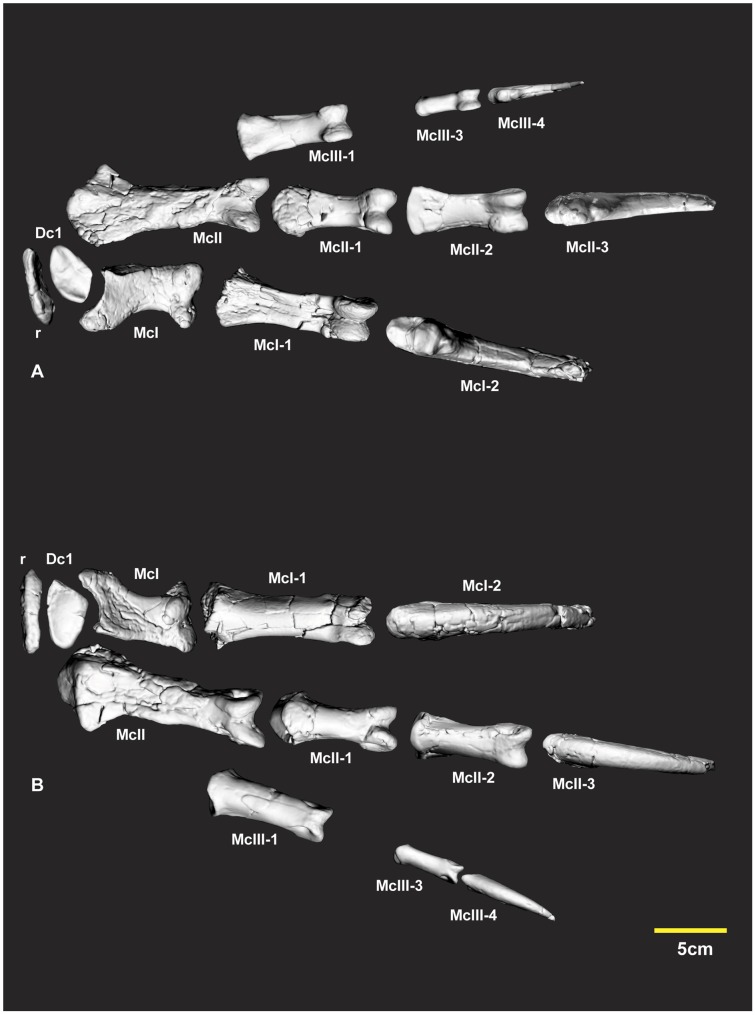
Articulated right manus elements. Computer generated specimen renders with modified clay surfaces to represent missing elements. Ventral view (A), dorsal view (B). *Abbreviations:* Dc1, distal carpal 1; McI, metacarpal I; McI-1, manual phalanx I-1; McI-2, manual phalanx I-2; McII, metacarpal II; McII-1, manual phalanx II-1; McII-2, manual phalanx II-2; McII-3, manual phalanx II-3; McIII-1, manual phalanx III-1; McIII-3, manual phalanx III-3; r, radiale.

The new phalanx specimens described in the following were initially identified with comparisons with *Allosaurus fragilis* reference to (See plates 43, 44, 45 in [11]). Several phalanges differ markedly from those of *Allosaurus*. The preservation of the *Australovenator* specimens enabled rearticulation with their corresponding phalanx. The articular facets vary to the degree that an incorrect articulation confirmed its incorrect placement within the manus (Figure 2). Three dimensional mirror printouts of specimens were used to clarify a phalanx position within the manus if its corresponding phalanx pertained from the opposite manus. Various cross-sections were figured to support specimen’s morphological descriptions reducing the need for lengthy descriptions, whilst still accurately describing and displaying distinct morphological characteristics.

### Humeri (Figures 3, 4 & 5AB)

**Figure 3:**
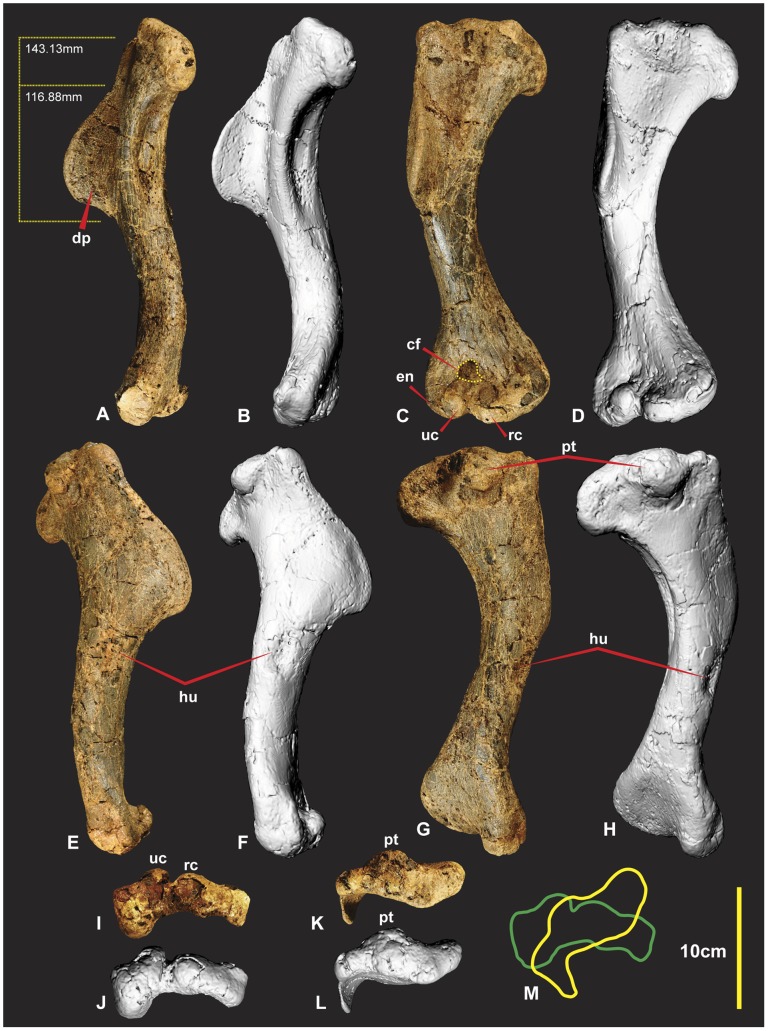
Right Humerus. Right Humerus in medial (A & B), dorsal (C & D), lateral (E & F), ventral (G & H), distal (I & J), proximal (K & L), outlines of proximal (yellow) and distal (green). *Abbreviations:* cf, concave facet; dp, deltopectoral crest; hu, attachment of humeroradialis muscle;uc,ulnar condyle;en, entepicondyle; pt, caudal tubicle; rc, radial condyle.

Both humeri are now known from the holotype (AODF 604). The right humerus is complete and excellently preserved. The left humerus sustained some damage to both distal and proximal ends. The humerus is a robust element with the proximal end measuring a third of the specimens’ entire length. The distal half of the shaft is bowed in medial view (Figure 3AB). The entire shaft appears bowed laterally in dorsal view (Figure 3CD). The humeral head is quadrilateral, with a distinctly pronounced tubercle on the posterior side (Figure 3EFGHKL). The deltopectoral crest is prominent, protruding around 40 mm from the main shaft. The deltopectoral crest is oriented at right angles to the proximal end and around 65–70 degrees to the distal end of the humerus. It is rounded and tapers more shallowly proximally than distally (Figure 5AB). Below the deltopectoral crest on the lateral side of the humerus is a large rounded scar for attachment of the humeroradialis muscle. In medial view the proximal portion of the shaft is deeply concave (Figure 3AB). There is a deep concave facet on the cranio-ventral side, proximal to the ulna and radial condyles (Figure 3C). The distal end is rotated outward from the plane of the proximal end. The entepicondyle is a small oval tubercle and is separated from the radial condyle by a deep groove. Measurements in Table S1.

**Figure 4:**
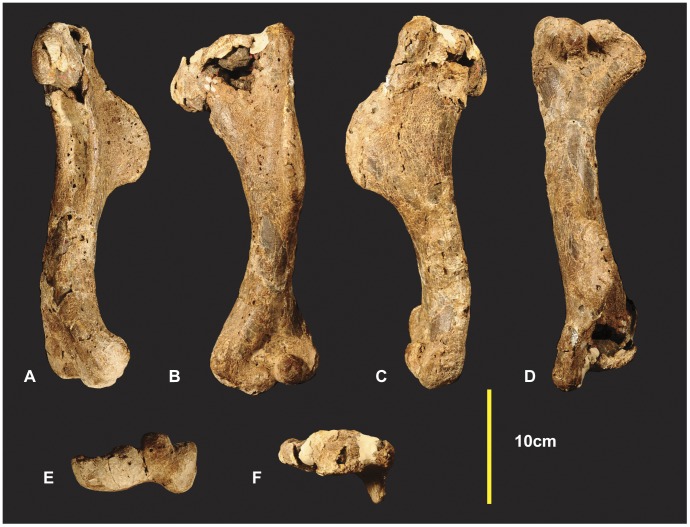
Left Humerus. Left Humerus in medial (A), dorsal (B), lateral (C), ventral (D), distal (E), proximal (F).

**Figure 5:**
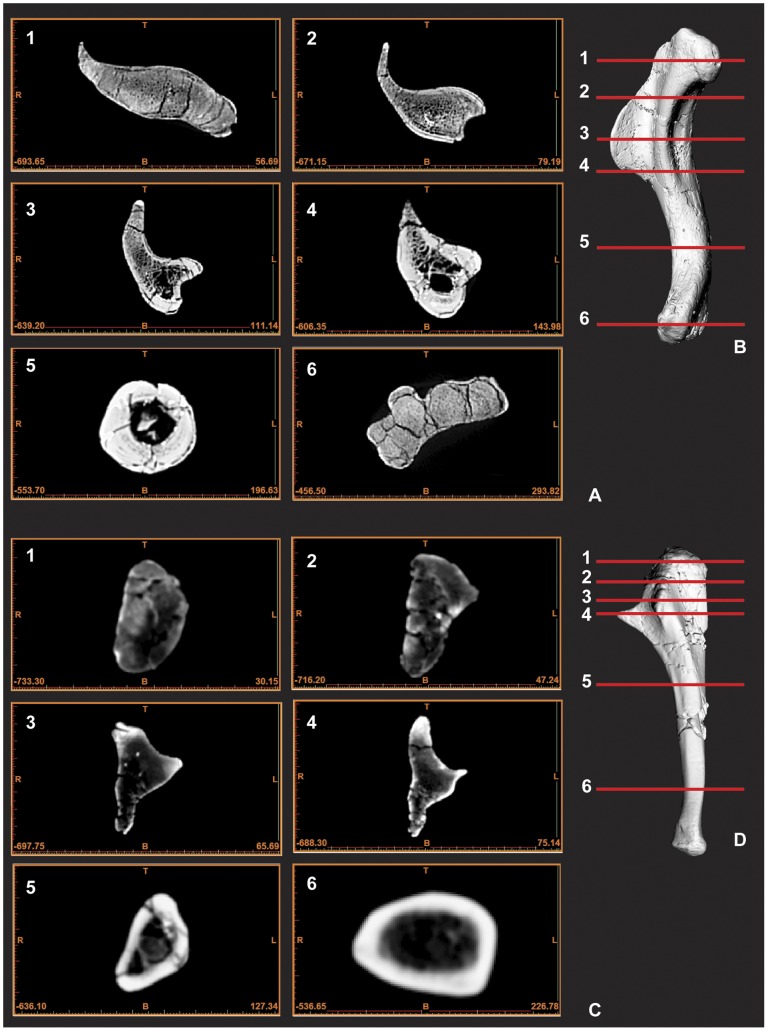
CT scan images of humerus and ulna displaying cross-section morphology. Humerus cross-sections images 1–6 (A), Computer generated render of humerus with corresponding image cross-section lines 1–6 (B), Ulna cross-sections images 1–6 (C), Computer generated render of ulna with corresponding image cross-section lines 1–6 (D). Scale is included as part of each CT image in mm.

### Left and Right Ulnae (Figures 5CD, 6 & 7)

Both ulnae were adequately described [1], however we have included improved figures, which include CT scan images to display the morphology of these elements more clearly. Measurements in Table S2.

**Figure 6:**
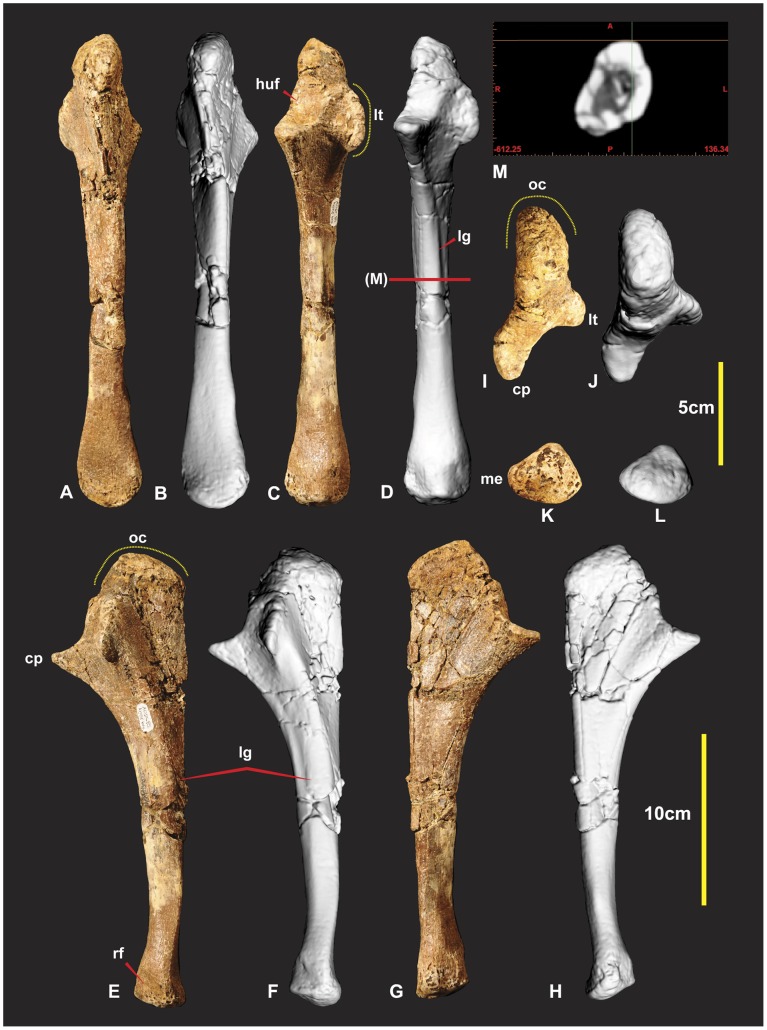
Right Ulna. Right Ulna in caudal (A & B), cranial (C & D), lateral (E & F), medial (G & H), proximal (I & J), distal (K & L). *Abbreviations:* cp, coronoid process; haf, humeral articular facet; lt, lateral tuberosity; me, medial expansion of the distal end; op, olecranon process; lg, lateral groove; rf, radial facet.

**Figure 7:**
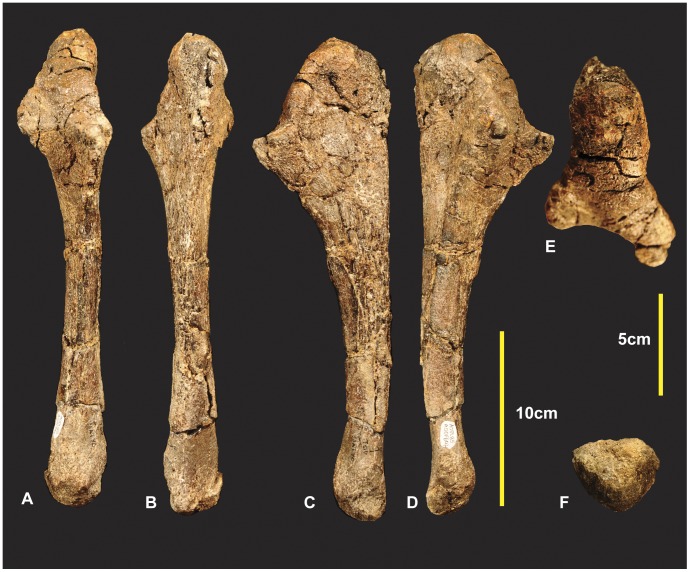
Left Ulna. Left Ulna in cranial (A), caudal (B), medial (C), lateral (D), proximal (E), distal (F).

### Left and Right Radii (Figures 8 & 9)

The left radius was adequately described [1] and is better preserved than the right which has sustained more fractures. The proximal end of the right radius is missing its articular surface and the distal end is missing the lateral margin of its lateral condyle. The radius is elongate with some lateral compression distorting the semicircular midshaft. Measurements in Table S3.

**Figure 8:**
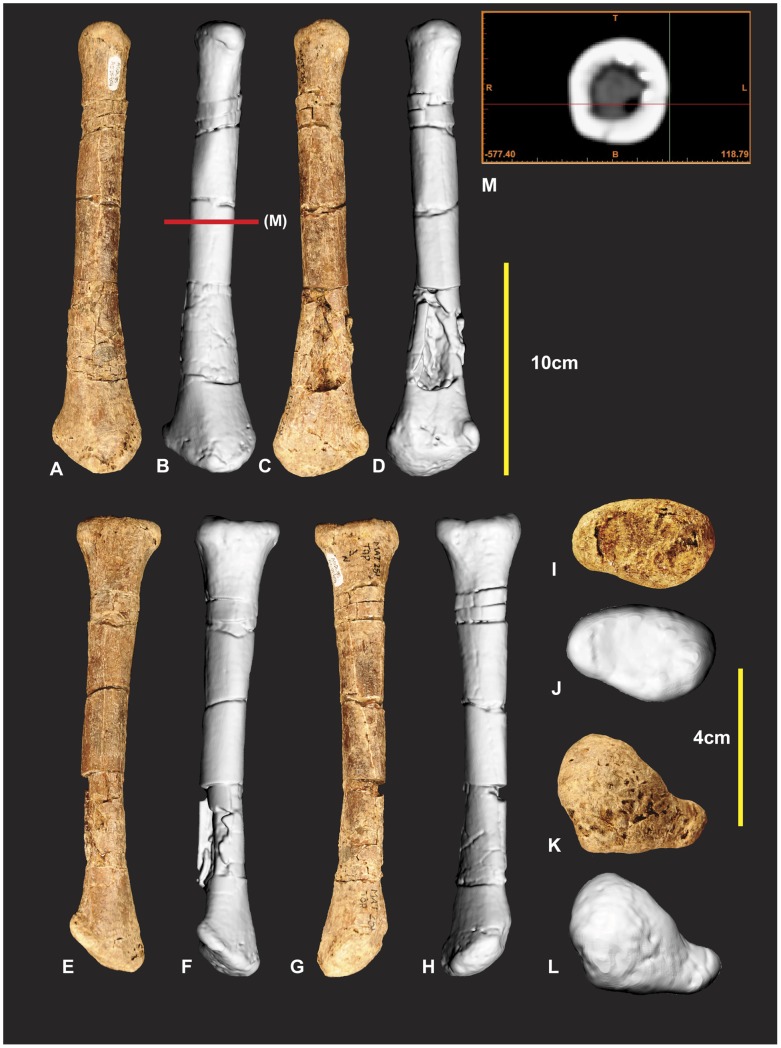
Left Radius. Left Radius in cranial (A & B), caudal (C & D), lateral (E & F), medial (G & H), proximal (I & J), distal (K & L) views, mid shaft CT image (M).

**Figure 9:**
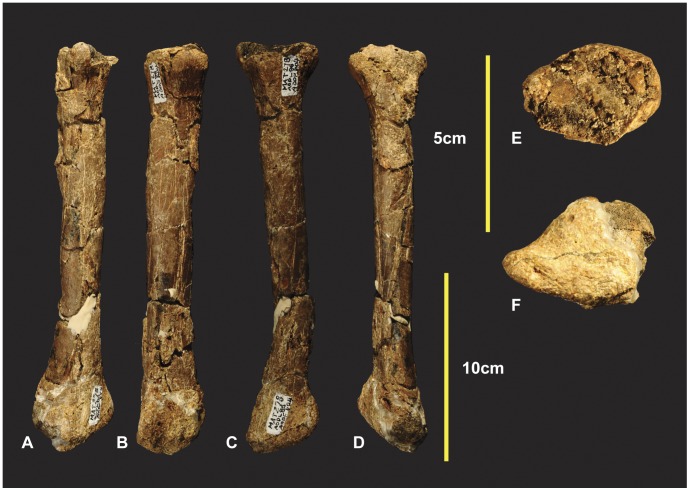
Right Radius. Right Radius in cranial (A), caudal (B), lateral (C), medial (D), proximal (E), distal (F) views.

### Right Radiale (Figure 10)

The right radiale is complete, oval in proximal aspect with the ventral margin slightly concave medially. The proximal surface is slightly rounded. The distal articular surface has a prominent convex bulge on the medial side. This thickened convex bulge tapers around the dorso-distal margin. This thickened dorso-distal margin borders a concave facet dorso-laterally. This facet pinches along the ventral margin which is the thinnest portion of the radiale. The distal surface perfectly articulates with distal carpal 1. Measurements in Table S4.

**Figure 10:**
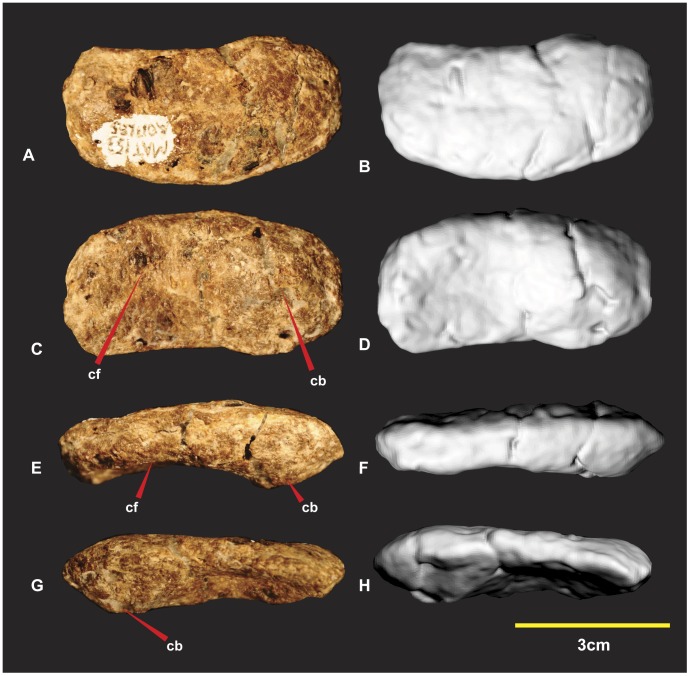
Right Radiale. Right Radiale in proximal (A & B), distal (C & D), dorsal (E & F), ventral (G & H). *Abbreviations:* cf, concave facet; cb, convex bulge.

### Right Distal Carpal 1 (Figure 11)

Various descriptions of this element referred it to either an intermedium or a distal carpal 1. Distal carpal 1 was used in a forearm description [12] of *Herrerasaurus ischigualastensis* [13] and was adopted for the same carpal buttressing the proximal end of metacarpal I and II in *Acrocanthosaurus atokensis* [14], [15]. In light of these descriptions we have also adopted the name distal carpal 1 for this element. Distal carpal 1 of *Australovenator* was not discovered in articulation with other carpals or metacarpals. Fortunately its preservation allowed it to fit perfectly with the proximal articular facet of metacarpal I and the distal articular facet of the radiale.

**Figure 11:**
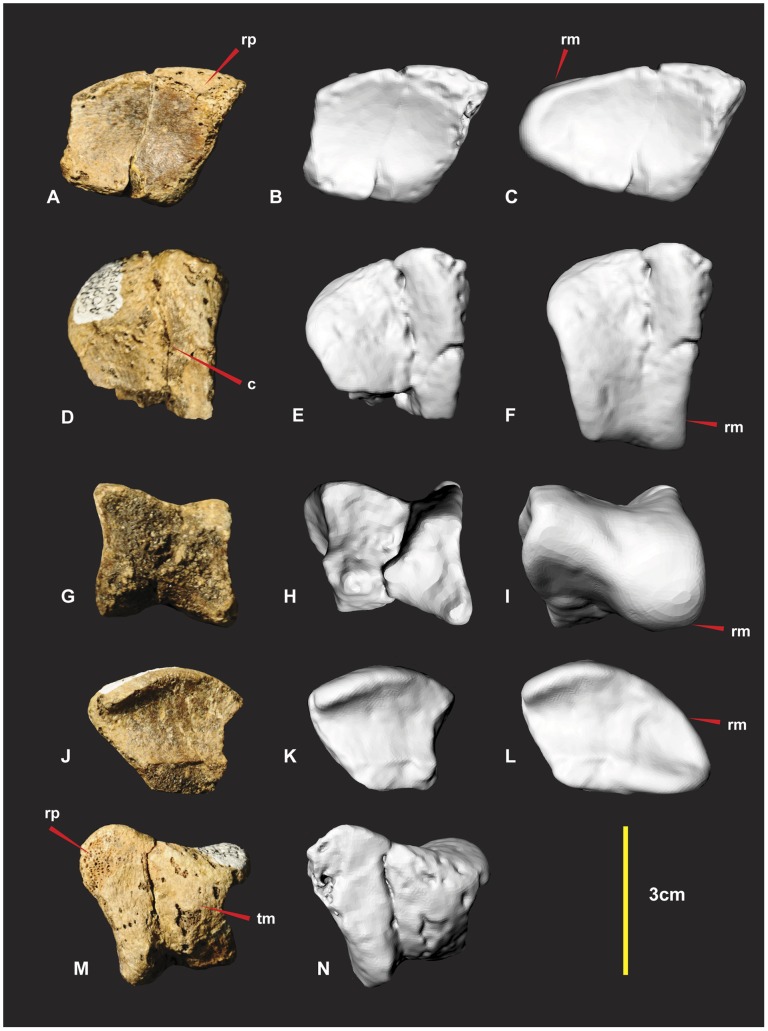
Right Distal Carpal 1. Right Distal Carpal 1 in dorsal (A, B & C), proximal (D, E & F), disto-lateral (G, H & I), ventral (J, K & L), disto-medial (M & N) views. Computer generated renders with modified clay surfaces to represent missing elements are displayed in (C, F, I & L) views. *Abbreviations:* c, convave region; rp, rugose and pitted areas; rm, repaired margin; tm, thin matrix covering rugose areas.

Unfortunately the majority of the carpal that articulated with metacarpal II was not preserved. The specimens’ surface is quite rugose and pitted. The surface is covered by a fine veneer of matrix. Sporadic gaps in this thin matrix reveal the porous rugose areas.

The dorsal (flexor) surface is concave and smooth. Its proximo-medial end is a rounded triangular shape, rugose and pitted (Figure 11A–C). The lateral margin that articulates with metacarpal II is missing but was suspected to have continued the smooth concave surface. The medial and disto-lateral margins were flat with the proximal margin tapering laterally appearing triangular. The outline of the proximal surface is rhombic and its surface is shallowly concave medially. In articulation with metacarpal I the concave surface tapers disto-ventrally and appears broadest medially (Figure 11D–F). The disto-lateral (flexor) surface is square. Despite the absence of the proximo-lateral margin, we can assume the morphology based on the preserved specimens’ contours and facet, preserved on the proximo-medial surface of metacarpal II (Figures 11GHI, 16D). The dorsal facet is thinner than the ventral facet from its articulation with metacarpal I. This feature appears to reverse disto-laterally as the dorsal facet expands and the ventral facet tapers. The entire surface is rugose and pitted. A shallow concave groove separates the convex facets medially. The ventral surface bears a broad mediolaterally orientated channel bounded by convex ridges. The disto-lateral margin extends to partially articulate with the proximal end of metacarpal II (Figure 11J–L). This feature guided a flexor tendon from the lower arm onto the wrist. The disto-medial (extensor) surface is concave and triangular (Figure 11MN). It articulates with the proximal articular facet of metacarpal I and partially articulates with the proximo-medial facet of metacarpal II. Two convex facets compose the disto-medial surface. The dorsal most facet is mostly flat but becomes pronounced laterally where it is separated from the ventral facet with a distinct groove. The ventral facet is much broader, shorter and rounded.

Distal carpal 1 is a robust element whose ventral surface channelled a flexor tendon from the lower arm that attached on a prominent notch on manual phalanx I-2. Measurements in Table S5.

### Right and Left Metacarpal I (Figure 12, 13)

Both left and right metacarpal I have been discovered. A poorly preserved left metacarpal I has been described [1]. Fortunately right metacarpal I (Figure 11) is nearly complete despite its numerous fractures it retains its’ original morphology.

**Figure 12:**
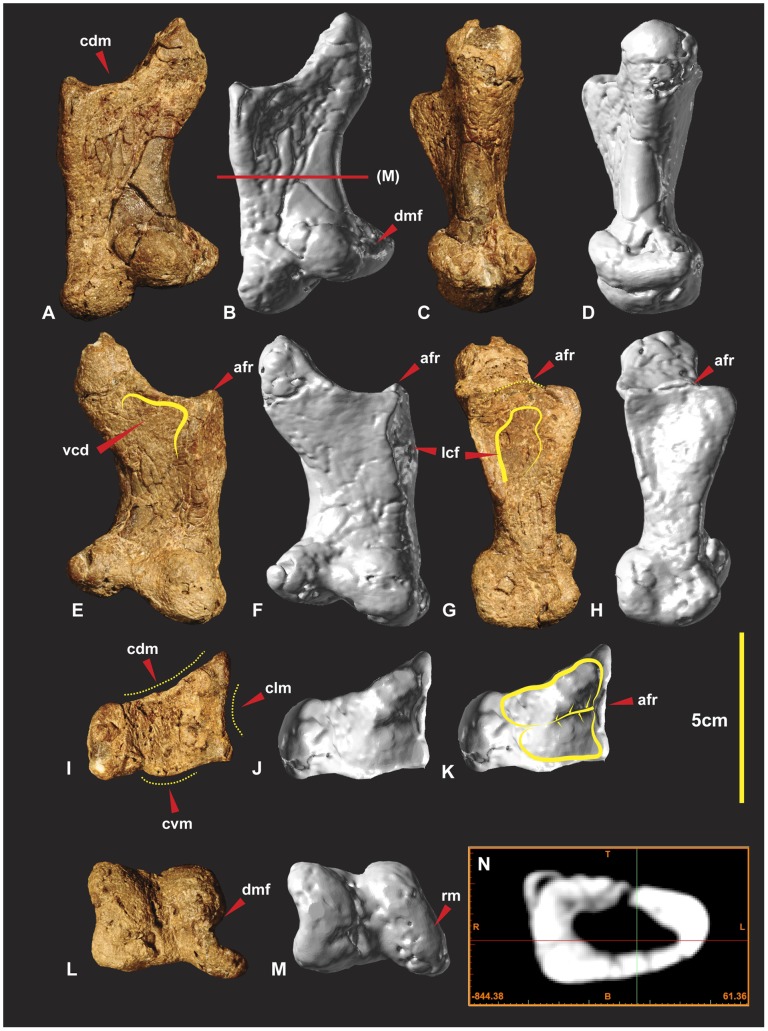
Right Metacarpal I. Right Metacarpal I in dorsal (A & B), medial (C & D), ventral (E & F), lateral (G & H), proximal (I & J), distal (K & L), mid shaft CT image (M). *Abbreviations:* afr, articular facet ridge; cdm, concave dorsal margin; clm, concave lateral margin; cvm, convex ventral margin; dmf, distal medial fossa; lcf, lateral concave facet; rm, repaired margin; vcd, ventral concave depression.

**Figure 13:**
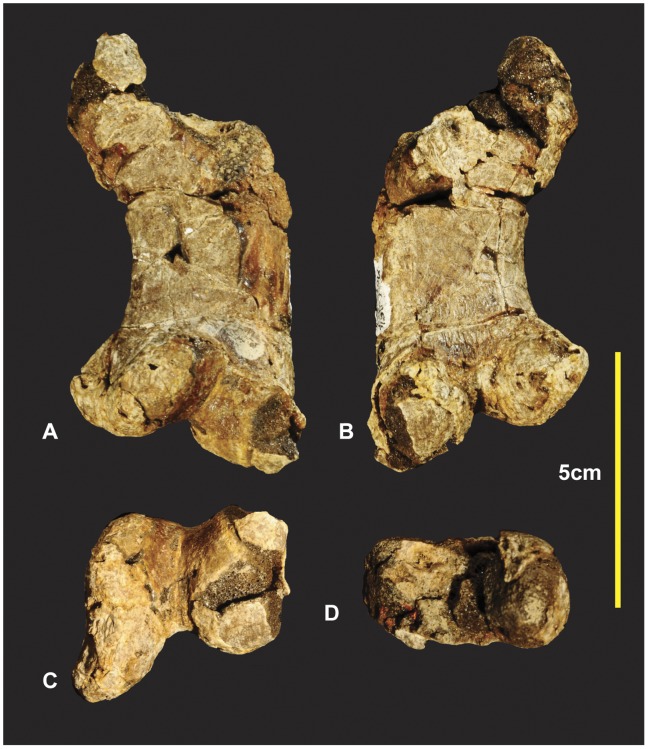
Left Metacarpal I. Left Metacarpal I in dorsal (A), medial (B), ventral (C), lateral (D), proximal (E), distal (F).

Right metacarpal I is a robust bone with a rounded triangular shaft, wide and flat on the lateral margin (Figure 12BM). The proximal articular facet is trapezoidal rather than triangular with a slightly rounded proximo-medial margin. The proximo-lateral margin is slightly concave in proximal view (Figure 12I–K). In lateral view this concave feature forms a prominent bordering dorso-ventrally orientated ridge that is taller medially (Figure 12E–H) in lateral view. This taller section forms a medio-lateral ridge that bisects the articular facet into two separate articular facets (Figure 12I–K). The dorsal facet is larger and deeper than the ventral facet (Figure 12K). The proximo-ventral margin is convex in proximal view (Figure 12I–K). In ventral view the proximo-ventral margin tapers into a slightly concave depression (Figure 12EF). The proximo-dorsal margin is slightly concave in both proximal and dorsal views (Figure 12ABIJK). A deep concave facet is present on the lateral face that occupies a proximo-ventral medio-ventral region. The deepest point of the facet is the proximo-ventral region. The proximo-dorsal region becomes convex (Figure 12E–H). The medial face is rounded and narrower than the lateral face, which is triangular in cross-section (Figure 12BCDN). The distal articular end is twisted counter clockwise toward the lateral margin. This twisted morphology exposes the distal medial fossa of the medial condyle so that it is visible in dorsal view (Figure 12AB). The articular end faces disto-medially. The medial distal condyle is taller than the lateral condyle and tapers distinctly outward dorso-ventrally from the distal aspect. The lateral condyle is slightly broader than the medial condyle (Figure 12LM). Measurements in Table S6.

### Left and Right Manual Phalanx I-1 (Figures 14, 15)

Both left and right manual phalanx I-1 have been briefly described and figured [1]. Here we identify that they were initially figured with the right actually pertaining to a left and vice versa.

**Figure 14:**
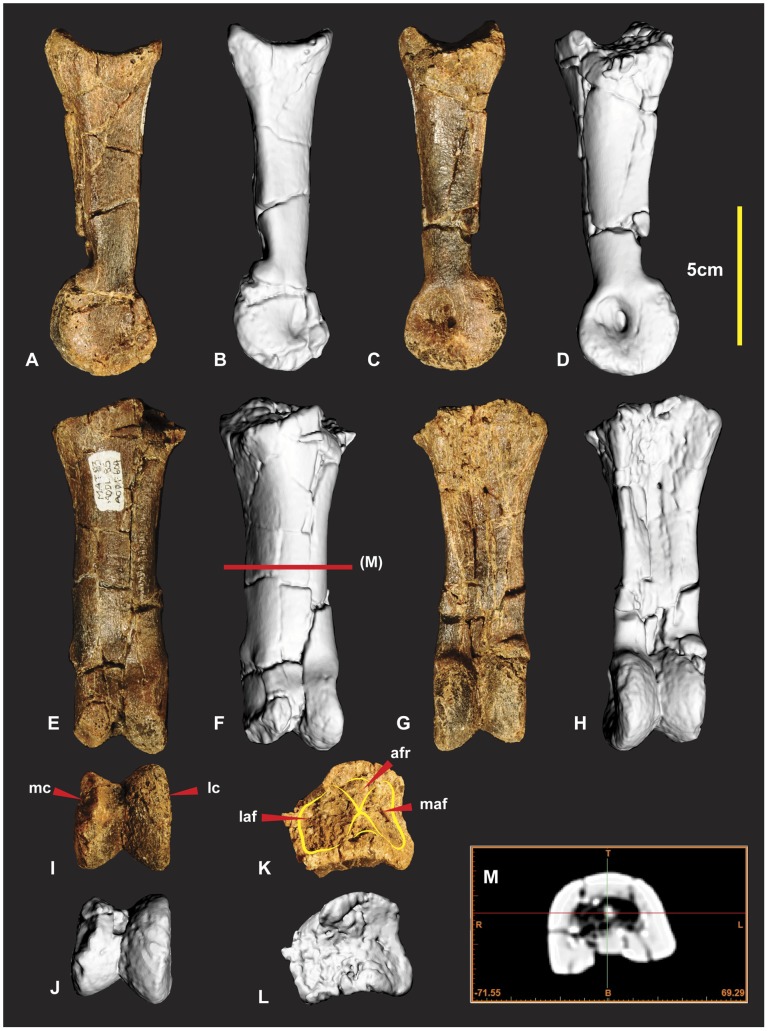
Left manual phalanx I-1. Left manual phalanx I-1 in medial (A & B), lateral (C & D), dorsal (E & F), ventral (G & H), distal (I & K), proximal (K & L) views, mid shaft CT image (M). *Abbreviations:* afr, articular facet ridge; laf, lateral articular facet; lc lateral condyle; maf, medial articular facet; mc, medial condyle.

**Figure 15:**
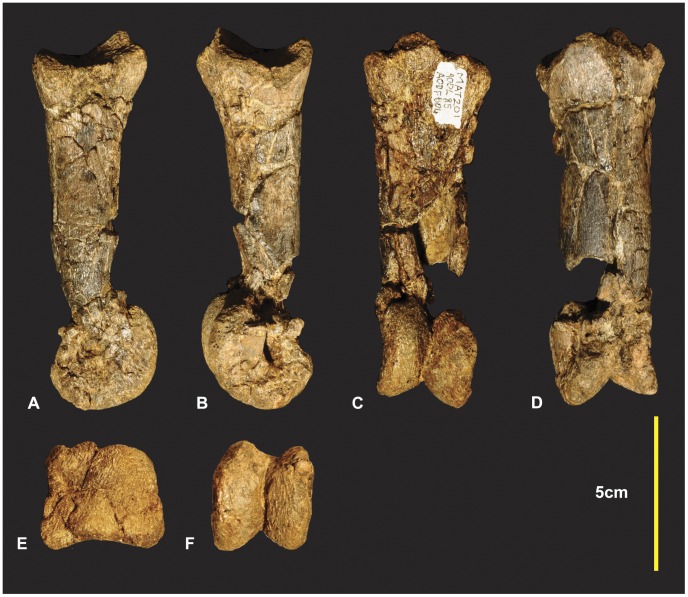
Right manual phalanx I-1. Right manual phalanx I-1 in lateral (A), medial (B), ventral (C), dorsal (D), proximal (E), distal (F) views.

The left McI-1 is mostly complete. The proximal articular surface of the left manual phalanxI-1 is divided into two articular facets by a dorso-ventrally orientated ridge (Figure 14KL). In lateral and medial views this ridge appears concave medially. The medial facet is shallower and taller than the lateral facet. The medial facet is rhombic with the dorsal margin wider than the ventral margin. The lateral facet is distinctly deeper than the medial facet. It is widest on its ventral margin and is triangular. A deep longitudinal depression is present along the ventral portion of the shaft. In ventral view the distal condyles appear slightly rotated clockwise (Figure 14GH). The lateral condyle is taller dorso-ventrally but shorter proximo-distally than the medial condyle. The right manual phalanx I-1 has a small section missing directly proximal to the lateral condyle. In lateral view the dorso-proximal and ventro-proximal margins of the condyle were not preserved (Figure 15). Measurements in Table S7.

### Right Manual Phalanx I-2 (Figure 16)

**Figure 16:**
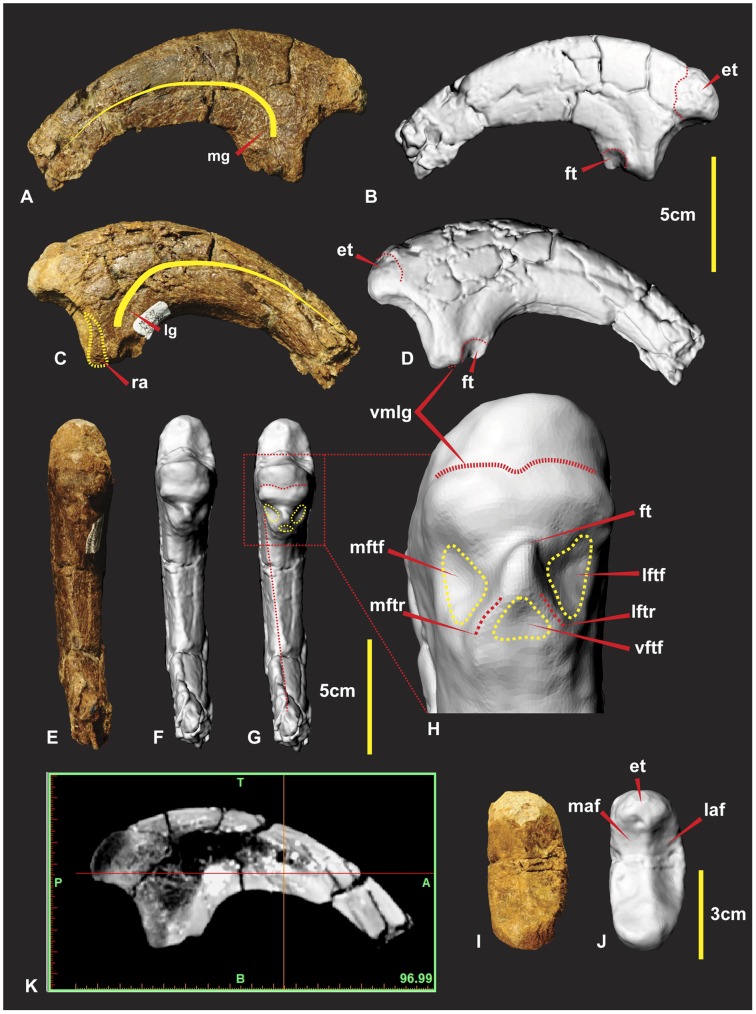
Right manual phalanx I-2. Right manual phalanx I-2 in lateral (A & B), medial (C & D), flexor tendon close up (E), ventral (F & G), proximal (H & I), central proximo-distal cross-section CT image (J). *Abbreviations:* et, extensor tubercle; ft, flexor tubercle; laf, lateral articular facet; lftf, lateral flexor tubercle facet; lftr, lateral flexor tubicle ridge; lg, lateral groove; maf, medial articular facet; mftf, medial flexor tubercle facet; mftr, medial flexor tubicle ridge; mg, medial groove; ra, rugose area; vmlg, ventral medio-lateral groove;;; vftf, ventral flexor tubercle facet.

Manual phalanx I-2 is the largest ungual of the manus. The specimen is both laterally and dorsally compressed. The distal end was broken during excavation and was reglued slightly out of alignment. A small portion of the distal end was not preserved. The facet as a whole is “deeply concave divided into two by a median ridge” [1]. The articular surface for manual phalanx I-1’s medial condyle has a greater dorso-ventral expression than the lateral condyle [1]. The lateral condyle is broader and deeper than the medial. The extensor tubercle is large, bulbous and tapers into the median ridge of the articular surface. A shallow ventral medio-lateral groove (vmlg) distinguishes the base of the articular facet (Figure 16DGH). In lateral view proximal to the ventral medio-lateral groove is a rugose area for ligament/tendon attachment (Figure 16C). On both the medial and lateral sides of the flexor tendon tubercle is concave semi-circular facets referred to as medial and lateral flexor facets. These ‘flexor facets’ accentuate the flexor tendon tubercle to a pronounced notch on the ventral surface (Figure 16GH).

Two small ridges taper medially and laterally from this notch enclosing the two concave semi-circular facets. A third triangular facet is directly distal to the prominent flexor tendon notch (Figure 16GH). A ventral crest originates from the medial side in a position just below the semicircular facet of the flexor tubercle. The crest is an extension of the medial surface which creates the concave ventral margin to the distal tip. The medial surface is mostly flat compared to the dorso-ventrally convex lateral surface [1]. The curve of the medial groove runs parallel to the ventral crest and tapers ventrally to the distal tip. The curve of the lateral groove runs parallel to the dorsal surface and tapers dorsally to the distal tip. Measurements in Table S8.

### Right Metacarpal II (Figure 17)

**Figure 17:**
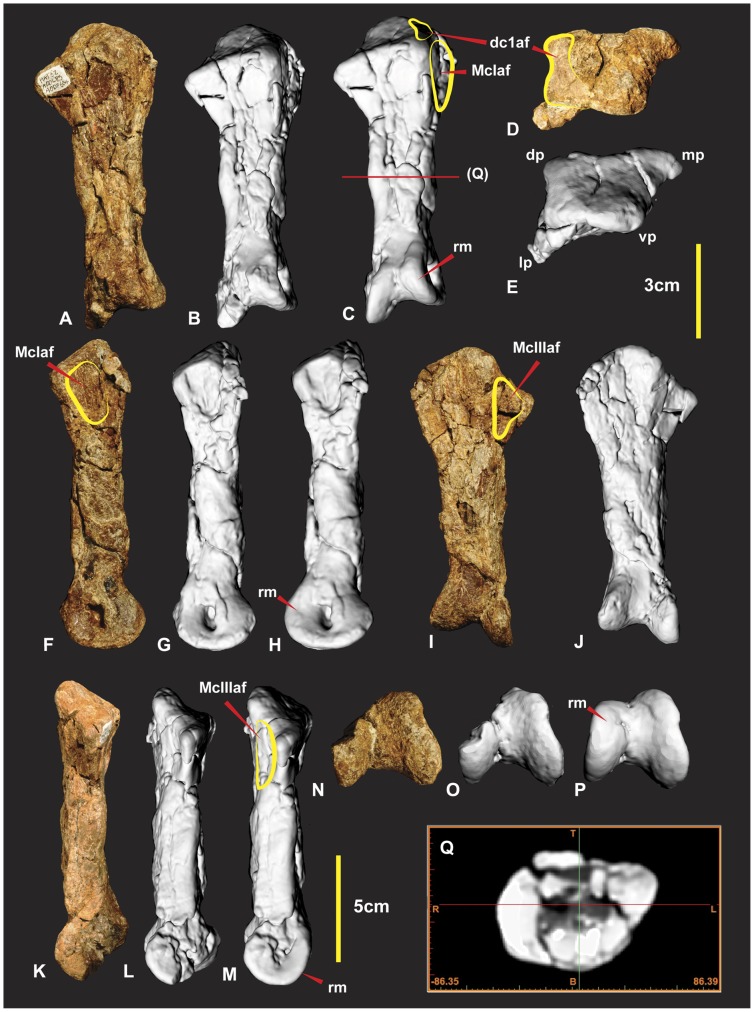
Right Metacarpal II. Right Metacarpal II in dorsal (A, B & C), proximal (D & E), medial (F, G & H), ventral (I & J), lateral (K, L & M), distal (N, O & P) views, mid shaft CT image (Q). Computer generated renders with modified clay surfaces to represent missing elements are displayed in (C, H, M & P) views. *Abbreviations:* dc1af, distal carpal 1 articular facet; dp, dorsal process; lp, lateral process; mp, medial process; McIaf, metacarpal I articular facet; rm, repaired margin; McIIIaf, metacarpal III articular facet; vp, ventral process.

Metacarpal II is approximately twice the length of metacarpal I. The metacarpal shaft is straight and is quadrangular in mid-shaft cross-section. The proximal end is transversely expanded, producing a medial crest which is located dorsal to metacarpal III. The proximal end is divided into four processes; dorsal, medial, lateral and ventral. The dorsal process is located on the medial side of the dorsal surface. The medial process extends medially on the medial surface. The lateral process extends laterally from the dorsal region dorsal surface. The ventral process extends proximal to the dorsal process and is located centrally between the medial and lateral processes. The ventro-lateral surface received the medial crest of metacarpal III (Figure 17HJM). The dorsal face of the medial process is shallowly concave. The dorsal process is low and rounded, bordered by the dorso-medial and dorso-lateral faces of the proximal end. In dorsal view the dorsal process extends low distally and is bowed medially. The medial process is less developed than the lateral and is narrow dorso-ventrally. It is boarded by the dorso-medial and ventro-medial surfaces. Both of these faces are concave and they form shallow fossae along the dorsal and ventral surfaces of the metacarpal shaft. The ventral process like the dorsal process is also low and rounded, bordered by the ventro-lateral and ventro-medial faces. The ventro-medial face has a shallow fossa which received the latero-medial crest from metacarpal I (Figure 17H). The distal end of the metacarpal is slightly expanded and divided into two distinct condyles. Both condyles are missing their dorsal margins. The medial condyle is estimated to be slightly larger than the lateral condyle. The internal groove is deep and broad. A deep ligament insertion pit is present on the medial margin of the medial condyle. Measurements in Table S9.

### Left Manual Phalanx II-1 (Figure 18)

This phalanx is near complete with only a small section missing from the proximal articular surface along the dorso-lateral margin. The distal medial condyle is missing a small fragment on its ventral margin. The phalanx is short and robust with a distinctly broad and tall proximal end. Its proximal height is just over half the length of the phalanx. The articular surface is separated into two articular facets. The medial facet is taller, narrower and distally deeper than the medial facet. The ventral intercondular process is proximally pronounced and forms a broad rounded subtriangular profile. In cross-section the shafts profile is D-shaped, rounded in the dorsal aspect and more flattened along the ventral aspect. In proximal aspect this ventral surface becomes offset creating the taller lateral margin of the articular facet. In ventral aspect the shaft is flattened from the proximal margin through to the mid shaft where it becomes more rounded directly proximal to the distal condyles. The distal condyles are relatively small in proportion to the proximal end. The lateral condyle is taller dorso-ventrally and appears slightly broader than the medial condyle. Measurements in Table S10.

**Figure 18:**
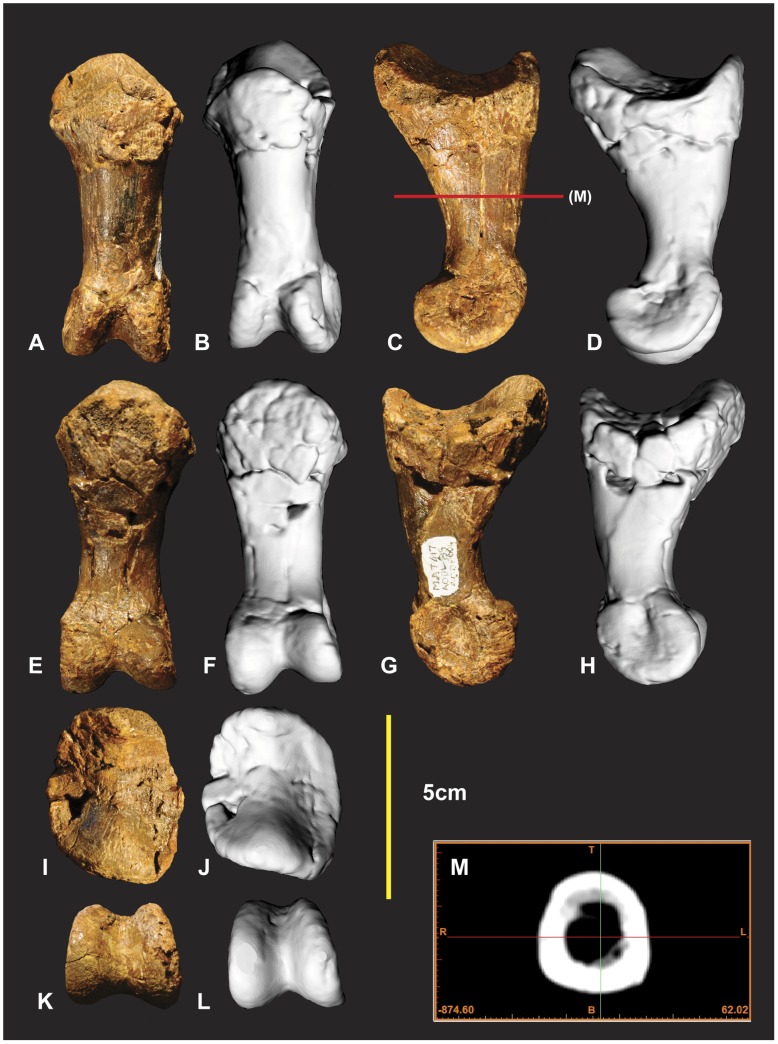
Left manual phalanx II-1. Left manual phalanx II-1 in dorsal (A & B), lateral (C & D), ventral (E & F), medial (G & H), proximal (I & J), distal (K & L), mid shaft CT image (M).

### Left Manual Phalanx II-2 (Figure 19)

The proximal and distal ends of this specimen were poorly preserved. The entire disto-lateral condyle is missing along with distal and ventral margins of the disto-medial condyle (Figure 18PQ). The proximal end retains portions of the articular facet. The medial ridge and surrounding proximal facet are missing. The proximal end is triangular (Figure 18M–O). The proximo-lateral articular facet is taller and slightly broader than the proximo-medial facet (Figure 18O). The proximo-medial facet appears slightly deeper proximo-distally than the proximo-lateral facet. The specimens’ shaft is predominately D shaped throughout the shaft. Measurements in Table S11.

**Figure 19:**
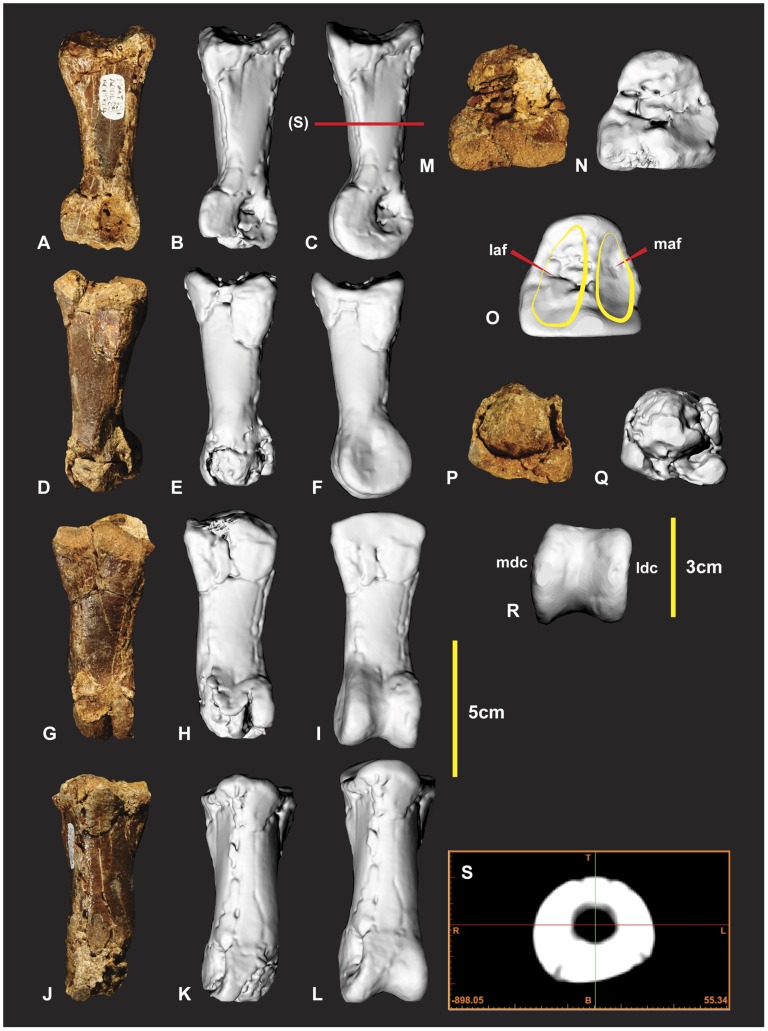
Left manual phalanx II-2. Left manual phalanx II-2 in medial (A, B & C), lateral (D, E & F), ventral (G, H & I), dorsal (J, K & L), proximal (M, N & O), distal (P, Q & R), mid shaft CT image (S). Computer generated renders with modified clay surfaces to represent missing elements are displayed in (C, F, I, L, O & R) views. *Abbreviations:* laf, lateral articular facet; ldc, lateral distal condyle; maf, medial articular facet; mdc, medial distal condyle.

### Right Manual Phalanx II-3 (Figure 20)

Manual phalanxII-3 is near equal size to manual phalanx I-2. The specimen is remarkably preserved retaining its original form with few fractures. The proximal end of the specimen was fractured around the dorsal side of the articular surface. Some additional minor fractures occur around the distal end of the claw. A small portion of the distal end was not preserved. The specimen is nearly symmetrical in all of its features except for it slight medial taper. The proximal articular facet is proportionally smaller in comparison to the articular facet of manusal phalanx I-2 but is similar morphologically to the third ungual initially described [1]. The proximal articular facet is broader medially and subtly tapers toward the dorsal and ventral surfaces. The lateral facet is deeper and slightly broader than the medial facet. A medial ridge divides the articular facet. The extensor tubercle is very similar to manual phalanx I-2 in being large, bulbous and tapering into the median ridge of the articular surface. A small ridge lips around the foundation of the articular facet surrounding the flexor tubercle similar to manual phalanx I-2. The ridge truncates dorsally into the vascular grooves on both the medial and lateral faces. This ridge separates the vascular grooves from the flexor tendon attachment area. Ventral to this ridge, a groove emphasizes both the rounded ridge and the flexor tendon attachment area. This groove tapers around the foundation of the articular facet to both the medial and lateral sides. Ventral to this groove, the flexor tendon attachment area appears bulbous and slightly tapers to a rounded point. The ventral surface of the ungual forms a rounded ellipsoid. Unlike manual phalanx I-2 the ventral surface is close to symmetrical. The medial and lateral grooves both trace the ventral surfaces contour. The grooves are close to symmetrical from the proximal to distal end. Both the lateral and medial surfaces are more rounded ventral to the vascular grooves than dorsal to them. These grooves are suspected to have supported a symmetrical sheath. Measurements in Table S12.

**Figure 20:**
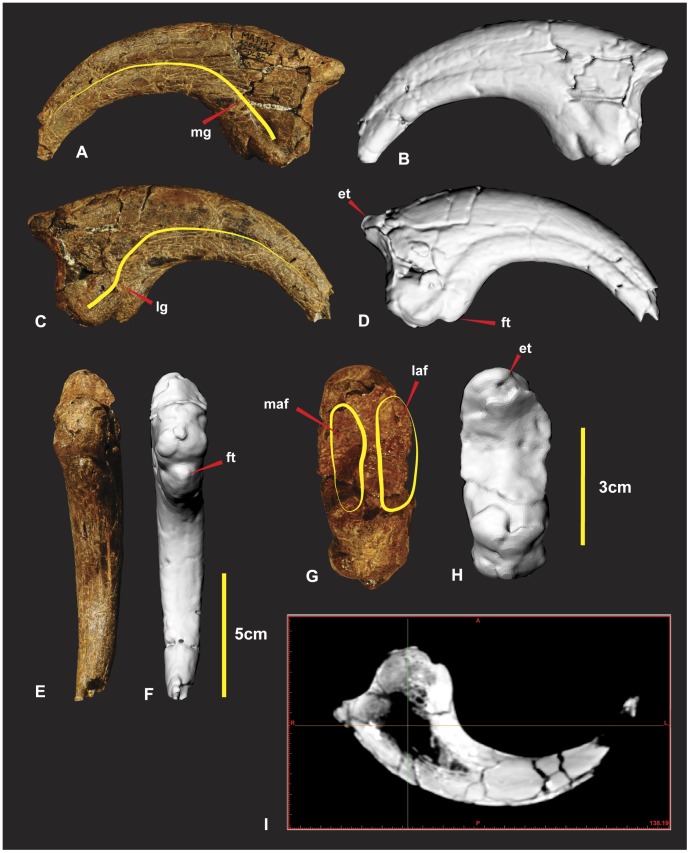
Right manual phalanx II-3. Right manual phalanx II-3 in medial (A & B), lateral (C & D), ventral (E & F), proximal (G & H), central proximo-distal CT image (I). *Abbreviations:* ex, extensor tubercle; ft, flexor tubercle; laf, lateral articular facet; maf, medial articular facet.

### Right Manual Phalanx III-1 (Figure 21)

**Figure 21:**
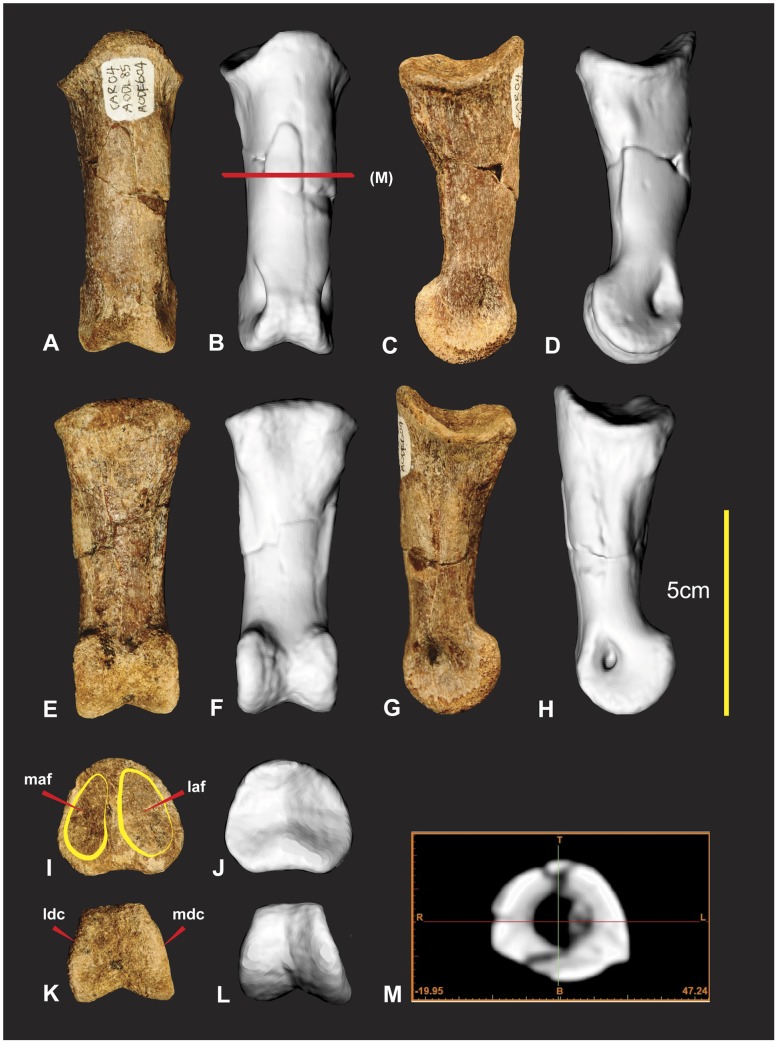
Right manual phalanx III-1. Right manual phalanx III-1 in dorsal (A & B), lateral (C & D), ventral (E & F), proximal (G & H), mid shaft CT image (M). *Abbreviations:* laf, lateral articular facet; ldc, lateral distal condyle; maf, medial articular facet; mdc, medial distal condyle.

This specimen was initially identified as manual phalanx II-2 [1]. The discovery of additional phalanges identified that it articulates with metacarpal III which is yet to be discovered. Following the discovery of manual phalanx II-3 it was clear that it was not robust enough to support such a large ungual. Slight morphological differences of the proximal and distal ends identify it as pertaining to the right manus. The proximal articular facet is triangular with a slightly concave ventral margin. The lateral articular facet is slightly taller, wider and deeper than the medial indicating that it is most likely a right phalanx (Figure 21IJ). The proximal end has a D-shaped cross-sectional outline that extends to midway along the shaft (Figure 21BM). The ventral surface is slightly rounded but flattens out to form medial and lateral ridges that taper into the shaft distally (Figure 21EF). The distal articular end is trapezoidal. The medial condyle is slightly taller and broader than the lateral condyle (Figure 21KL). Measurements in Table S13.

### Right and Left Manual Phalanx III-3 (Figure 22, 23)

Both left and right manual phalanx III-3 specimens have been discovered. The right was previously described [1] and articulated with the right manual phalanx III-4. The medial condyle is taller than the lateral. The proximal articular facet is deeper and slightly taller on the medial side compared with the lateral side. Measurements in Table S14.

**Figure 22:**
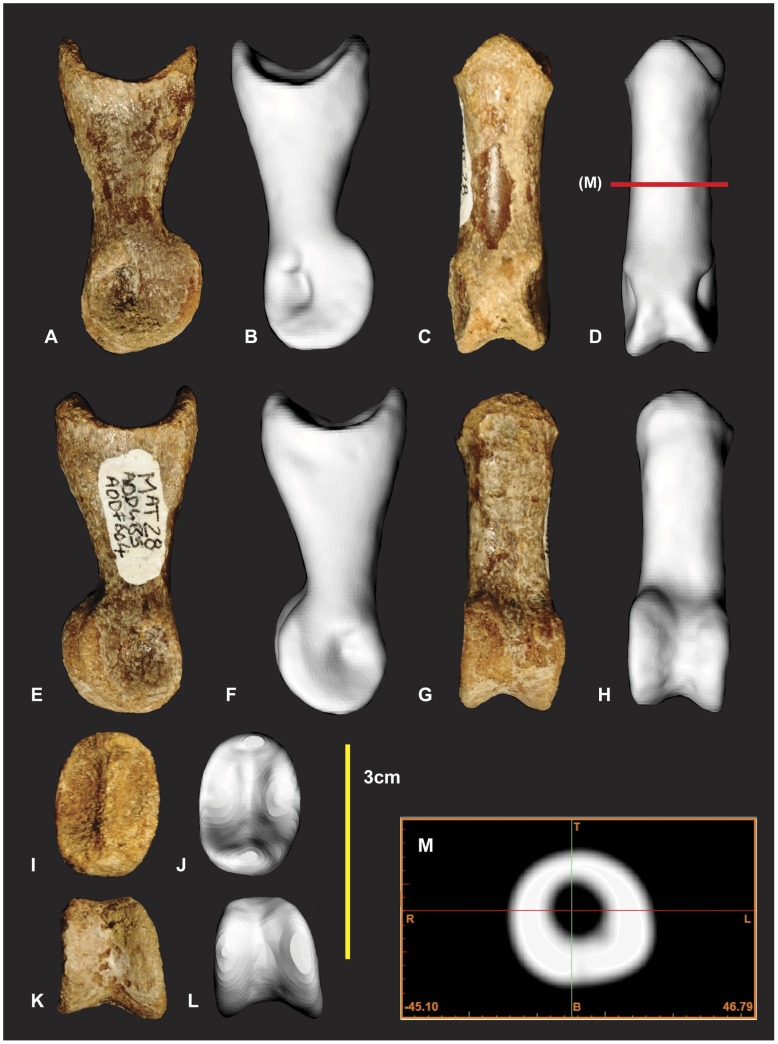
Right manual phalanx III-3. Right manual phalanx III-3 in medial (A & B), dorsal (C & D), lateral (E & F), ventral (G & H), mid shaft CT image (I).

**Figure 23:**
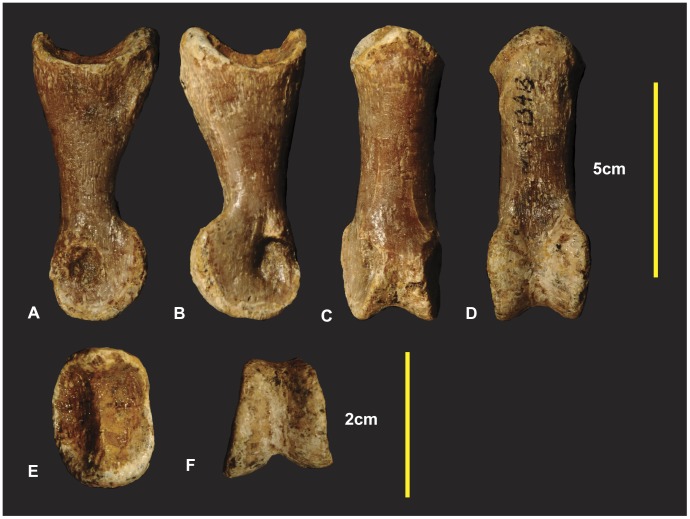
Left manual phalanx III-3. Left manual phalanx III-3 in medial (A), lateral (B), dorsal (C), ventral (D), proximal (E) and distal (F) views.

### Right Manual Phalanx III-4 (Figure 24)

**Figure 24:**
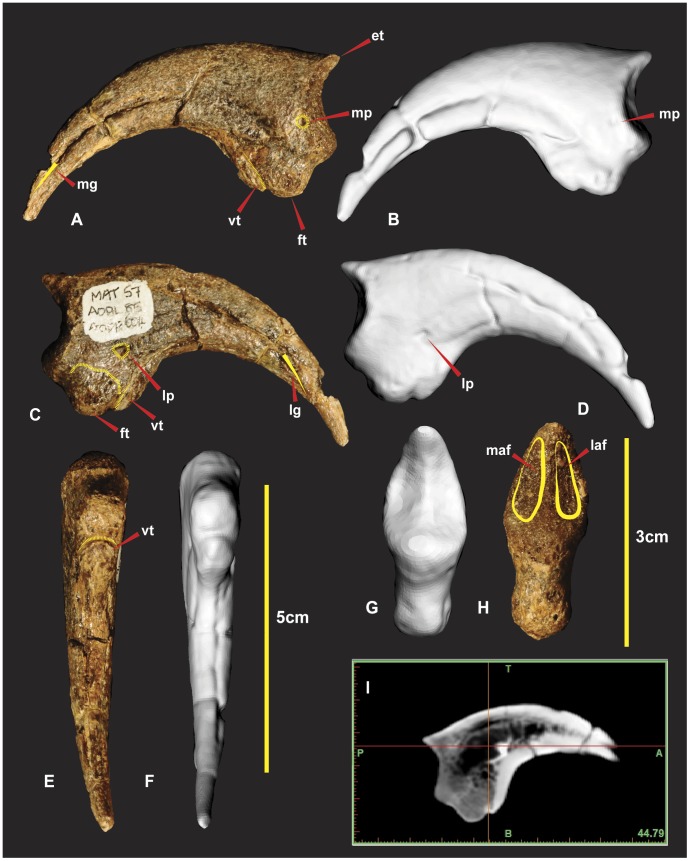
Right manual phalanx III-4. Right manual phalanx III-4 medial (A & B), lateral (C & D), ventral (E & F), proximal (G & H), central proximo-distal CT image (I).

Manual phalanx III-4 is the smallest manual ungual and is remarkably preserved, retaining its original form with very few fractures. A small chip is missing on the dorsal surface toward the distal end. A distal fracture has resulted in minor loss which possibly occurred during excavation. Another fracture occurs on the lateral side from the distal end through to the top of the dorsal surface. The ungual tapers distally, towards the lateral side (Figure 24F). The proximal articular facet is broadest medially, subtly tapering toward the dorsal and ventral surfaces. The ventral portion of the articular facet remains broader than the dorsal portion. The articular surface for the medial condyle has greater dorso-ventral expression than the lateral condyle (Figure 24GH) [1]. The extensor tubercle is small in comparison to manual unguals I and II. Ventral to the articular facet, the proximal end of the ungual is slightly concave and merges into a prominent bulbous flexor tubercle, closely resembling manual phalanx II-3. Both the medial and lateral sides of the flexor tubercle are rugose and pitted, presumably for the flexor tendon attachment, with the lateral being more pitted than the medial (Figure 24). Interestingly these pits are not visible in the manual phalanx I-2 ungual. The medial surface is rounded compared to the more subtle, flatter lateral surface. On the medial surface a small pit is located centrally between the flexor and extensor tubercles directly distal to the articular facet (Figure 24AB). On the lateral surface a small pit is located at the proximal end of the lateral groove (Figure 24CD). The medial groove originates from a medial position at the proximal end of the ungual and migrates to the dorsal surface distally (Figure 24A). The lateral groove originates from a similar proximal position to the medial groove and migrates to the ventral surface at the distal end (Figure 24C). Ventral to the flexor tubercle is a smaller tubercle delineated by medial and lateral grooves (Figure 24ACE). Measurements in Table S15.

### Neovenatorid Comparisons

A new clade Neovenatoridae was established on the basis of synamorphies shared between Neovenator salerii [16], Aerosteon riocoloradensis [17], Chilantaisaurus tashuikouensis [18], Fukuiraptor kitadaniensis [19], Megaraptor namumhuaiquii [20], Orkoraptor burkei [21] and Australovenator wintonensis [1]–[2]. The new forearm and manus specimens described of the holotype specimen Australovenator wintonensis enabled additional comparisons with known neovenatorid taxa. Comparisons were only made using available published illustrations and descriptions, therefore some characteristics although potentially present were not effectively available for comparison due to the poor detail in figures or absence of particular views. In these cases the direct examination of specimens would be preferred to identify diagnostic features to be used in future cladistical analysis. Measurements in Table S16.

### Humerus

*Fukuiraptor* and *Chilantaisaurus* are the only other neovenatorid taxa in which the humerus was preserved. The morphology of the humerus in *Fukuiraptor* is similar to *Australovenator* (see Figure 7 in [19]). The shaft displays a similar bowed morphology. The deltopectoral crest is rounded and tapers both proximally and distally although is slightly more abrupt distally. The distal end appears rotated outward from the plane of the proximal end. The distal end of the *Fukuiraptor* humerus is has two condyles separated by an intercondyler groove. The radial condyle is described as twice the size as the ulna condyle [19]. In *Australovenator* the ulna condyle is dorso-ventrally taller than the radial condyle in distal view (Figure 3IJ). Medio-laterally the radial condyle is slightly wider than the ulna condyle. Therefore the description identifies a subtle difference between the morphology of the distal ends between the two specimens.

The humerus of *Chilantaisaurus* is considerably more robust and differs markedly from *Australovenator* (see Figure 1 in [22]). The shaft is straight from the proximal portion of the deltopectoral crest to the distal end of the shaft, compared with the previously described bowed shaft of *Australovenator*. The deltopectoral crest of *Chilantaisaurus* projects from the shaft at close to 90 degrees at both its proximal and dorsal limits, which is unlike the tapered morphology shared between *Fukuiraptor* and *Australovenator*. It lacks the distinctly pronounced tubercle on the caudal side possessed by *Australovenator*.

Two morphological comparisons used in the erection of Neovenatoridae (see Appendix S1 in [2]), was the humerus/femur ratio and the humerus deltopectoral crest/humeral length ratio. The humerus/femur ratio (0.56) of *Australovenator* supports its placement within Neovenatoridae [20]. Two deltopectoral crest lengths are here used to calculate the humerus deltopectoral crest/humeral length ratio as it is unclear how the deltopectoral crest was measured in [20]. The first deltopectoral crest length was its extent along the shaft 116.88 mm. The second length extended to the proximal end of the humerus 143.13 mm (Figure 3A). The respective ratios were 0.38 and 0.47. Given the length of the deltopectoral crest is determined from the proximal end of the humerus the resulting ratio of 0.47 proves to be a synapomorphy for Neovenatoridae (Table S1).

### Manual Phalanx II-1

The manual phalanx II-1 from *Fukuiraptor* (see Figure 9 in [19]) is markedly different to the manual phalanx II-1 of *Australovenator*. The dorso-ventral height of the distal condyles and the proximal end are near equivalent in height. In *Australovenator* the distal condyles dorso-ventral height is markedly smaller compared to the dorso-ventral proximal height (add ratio). In dorsal view (see Figure 9B [19]) the phalanx shaft is slightly bowed laterally. *Australovenator* has a relatively straight shaft proximo-distally. In ventral view (see Figure 9C [19]) the phalanx appears ventrally concave. This concave feature is not present in the *Australovenator* manual phalanx II-1.

### Manual Phalanx I-2

A manual phalanx of *Chilantaisaurus* was identified as I-2 (see Figure 2 in [22]). Its morphology more closely resembles manual phalanx II-3 of *Australovenator*. The photographed *Chilantaisaurus* specimen displays an ungual that appears more symmetrical on both the medial and lateral sides compared with the asymmetrical morphology of the *Australovenator* specimen. The medial and lateral grooves also appear to follow a symmetrical trace from the proximal end through to the distal tip. The proximal view of the specimen indicates a slight bulge on the medial side which is most likely resulting from the specimen being slightly crushed on the lateral side. These morphological features suggest the *Chilantaisaurus* ungual belongs to the second digit.

The *Fukuiraptor* specimen (see Figure 10–A–D in [19]) appears to have a smaller arc length than that of *Australovenator*. This comparison is drawn from the figured sketches. The *Fukuiraptor* specimen is crushed at the proximal end which may give the appearance of a more stunted arc length. The medial and lateral grooves appear asymmetrical as demonstrated in cross-section drawings. Like *Australovenator* the lateral groove tapers dorsally and the medial groove tapers more centrally towards the distal tip (see Figure 10D in [19]). Manual phalanx I-2 is only slightly larger than manual phalanx II-3 in *Fukuiraptor* which is similar in *Australovenator*.

The sketched illustrations of *Megaraptor* elements display a close morphology to *Australovenator*. Initially the manus ungual of *Megaraptor* was described as a retractable second digit pedal ungual belonging to either a dromaeosaurid or a troodontid [20]. Its re-identification from a pedal ungual to the first manual was rectified [23] however the initial diagrams (see Figure 3A–E in [20]) display the morphological features in greater detail than the later publication (compare with Figure 9A in [23]). The illustration reveals the presence of a pronounced flexor tubercle and directly proximal to this the morphology appears similar to that of *Australovenator*.

The proportional size of the manual phalanx I-2 compared with manual phalanx II-3 in *Megaraptor* is considerably greater than those of *Australovenator* and *Fukuiraptor* (see Figure 9A in [23]).

### Manual Phalanx II-3

The description of the manual phalanxII-3 of *Fukuiraptor* (see Figure 10E–G [19]) indicates close similarity to that of *Australovenator*. It differs in what appears to be a shorter arc length. Just distal to the flexor tubercle, the ventral portion of the ungual appears more strongly recurved than the *Australovenator* specimen. The near symmetrical morphology of this ungual is shared between *Fukuiraptor*, *Megaraptor*, *Australovenator* and possibly *Chilantaisaurus.*

### Manual Phalanx III-4

Manual phalanx III-4 of *Megaraptor* is incomplete and no comparisons could be made with the diagrams available (see Figure 9C in [23]). The description states that this ungual was devoid of a lateral groove [23]. Interestingly the same ungual in *Australovenator* possesses a lateral groove although it is less prominent than the medial groove.

The proximal height and width ratios of the manus unguals were used as neovenatorid synapomorphies in that if the ratio was >2.3 it supported placement within Neovenatoridae. The ratios for *Australovenator*, manual phalanx I-2, manual phalanx II-3 and manual phalanx III-4 are 2.29, 2.22 and 2.41 respectively.

## Discussion

The preserved forearms of *Australovenator wintonensis* are the most complete so far discovered within neovenatorids. The morphological comparisons of the humerus, manual phalanx I-2, manual phalanx II-1, manual phalanx II-3 and manual phalanx III-4 with other published neovenatorid specimens identified characteristics that could be used for future phylogentic analysis. Such characteristics include a comparison of the distal end of the humerus focusing on the size of the ulna and radial condyles; the morphology of the humeral deltopectoral crest; ungual arc lengths and the orientation of the medial and lateral grooves. The morphology of the manus phalanges are difficult to compare amongst neovenatorids as the exact skeletal identification of manus phalanes are questionable in specimens without corresponding phalanx. One such example was the initial description of manual phalanx II-2 of *Australovenator wintonensis* [1] that was redescribed within this manuscript as manual phalanx III-1. The near complete forearm of *Australovenator* can be used as a direct comparison with other neovenatorids to properly assign the preserved phalanx to their correct manus position. A biomechanical investigation is currently being undertaken to compare the functional morphology with other theropod taxa.

## Supporting Information

Table S1 **Humeri measurements.**
(DOC)

Table S2 **Ulnae measurements.**
(DOC)

Table S3 **Radii measurements.**
(DOC)

Table S4 **Radiale measurements.**
(DOC)

Table S5 **Distal carpal 1 measurements.**
(DOC)

Table S6 **Metacarpal 1 measurements.**
(DOC)

Table S7 **Manual phalanx I-1 measurements.**
(DOC)

Table S8 **Manual phalanx I-2 measurements.**
(DOC)

Table S9 **Metacarpal II measurements.**
(DOC)

Table S10 **Manual phalanx II-1 measurements.**
(DOC)

Table S11 **Manual phalanx II-2 measurements.**
(DOC)

Table S12 **Manual phalanx II-3 measurements.**
(DOC)

Table S13 **Manual phalanx III-1 measurements.**
(DOC)

Table S14 **Manual phalanx III-3 measurements.**
(DOC)

Table S15 **Manual phalanx III-4 measurements.**
(DOC)

Table S16 **Neovenatorid measurements.**
(DOC)
