# Supplementary material for: New Forearm Elements Discovered of Holotype Specimen Australovenator wintonensis from Winton, Queensland, Australia
Source: PLoS One. 2012 Jun 27;7(6):e39364. doi: 10.1371/journal.pone.0039364 (PMC3384666; doi:10.1371/journal.pone.0039364)
Supplement: Table S1 — Humeri measurements. (DOC) [file pone.0039364.s001.doc]

Table S1: Humerus measurements (mm)

|  | Left | Right |
| --- | --- | --- |
| Length (proximo-distal) | 307.35 | 303.35 |
| Distal transverse width | 94.4 | 95.14 |
| Proximal transverse width | 110.33 | 101.79 |
| Mid-shaft transverse width | 47.02 | 44.2 |
