# Supplementary material for: New Forearm Elements Discovered of Holotype Specimen Australovenator wintonensis from Winton, Queensland, Australia
Source: PLoS One. 2012 Jun 27;7(6):e39364. doi: 10.1371/journal.pone.0039364 (PMC3384666; doi:10.1371/journal.pone.0039364)
Supplement: Table S2 — Ulnae measurements. (DOC) [file pone.0039364.s002.doc]

Table S2: Ulna measurements (mm)

|  | Left | Right |
| --- | --- | --- |
| Length (proximo-distal) | 265.58 | 267.22 |
| Distal transverse width | 36.78 | 35.96 |
| Distal height (dorso-ventral) | 28.99 | 28 |
| Proximal transverse width | 36.88 | 39.9 |
| Proximal height (dorso-ventral) | 75.48 | 74.3 |
| Mid-shaft transverse width | 18.94 | 18.72 |
