# Supplementary material for: New Forearm Elements Discovered of Holotype Specimen Australovenator wintonensis from Winton, Queensland, Australia
Source: PLoS One. 2012 Jun 27;7(6):e39364. doi: 10.1371/journal.pone.0039364 (PMC3384666; doi:10.1371/journal.pone.0039364)
Supplement: Table S3 — Radii measurements. (DOC) [file pone.0039364.s003.doc]

Table S3: Radius measurements (mm)

|  | Left | Right |
| --- | --- | --- |
| Length (proximo-distal) | 215.37 | 211.28 |
| Distal transverse width | 32.04 | 28.39 |
| Distal height (dorso-ventral) | 42.8 | 44.07 |
| Proximal transverse width | 43.58 | 41.69 |
| Proximal height (dorso-ventral) | 29.33 | 26.2 |
| Mid-shaft transverse width | 21.32 | 21.11 |
