# Supplementary material for: New Forearm Elements Discovered of Holotype Specimen Australovenator wintonensis from Winton, Queensland, Australia
Source: PLoS One. 2012 Jun 27;7(6):e39364. doi: 10.1371/journal.pone.0039364 (PMC3384666; doi:10.1371/journal.pone.0039364)
Supplement: Table S4 — Radiale measurements. (DOC) [file pone.0039364.s004.doc]

Table S4: Radiale measurements (mm)

|  | Right |
| --- | --- |
| Length (medio-lateral) | 52.44 |
| Length (dorso-ventral) | 24.92 |
