# Supplementary material for: New Forearm Elements Discovered of Holotype Specimen Australovenator wintonensis from Winton, Queensland, Australia
Source: PLoS One. 2012 Jun 27;7(6):e39364. doi: 10.1371/journal.pone.0039364 (PMC3384666; doi:10.1371/journal.pone.0039364)
Supplement: Table S5 — Distal carpal 1 measurements. (DOC) [file pone.0039364.s005.doc]

Table S5: Right Distal Carpal 1 measurements (mm)

|  | Actual specimen | Estimated specimen length |
| --- | --- | --- |
| Dorso-medial margin | 28.11 |  |
| Dorsal width (medio-lateral) | 28.1 | 38.31 |
| Dorsal medial width (proximo-distal) | 25.03 |  |
| Ventro-medial margin | 18.75 |  |
| Ventral width (medio-lateral) | 20.79 | 34.51 |
| Ventro-lateral margin | 14.64 | 25.54 |
| Dorso-ventral width | 22.23 |  |
| Cranio-caudal height medial side | 34.6 |  |
| Cranio-ventral height centre | 22.62 |  |
| Cranio-ventral height lateral side |  | 23.8 |
