# Supplementary material for: New Forearm Elements Discovered of Holotype Specimen Australovenator wintonensis from Winton, Queensland, Australia
Source: PLoS One. 2012 Jun 27;7(6):e39364. doi: 10.1371/journal.pone.0039364 (PMC3384666; doi:10.1371/journal.pone.0039364)
Supplement: Table S6 — Metacarpal 1 measurements. (DOC) [file pone.0039364.s006.doc]

Table S6: Right Metacarpal 1 measurements (mm)

| Medial length (proximo-distal) | 79.91 |
| --- | --- |
| Lateral length (proximo-distal) | 72.05 |
| Proximal width | 40.77 |
| Proximal width ventral margin | 39.16 |
| Proximal width dorsal margin | 47.5 |
| Proximal height dorso-ventral margin | 32.81 |
| Distal width (dorsal) | 32.98 |
| Distal width (ventral) | 44.04 |
| Lateral condyle height | 27.79 |
| Medial condyle height | 35.89 |
| Mid-shaft width dorsal | 33.15 |
| Mid-shaft width ventral | 28.89 |
| Mid-shaft height | 22.07 |
