# Supplementary material for: New Forearm Elements Discovered of Holotype Specimen Australovenator wintonensis from Winton, Queensland, Australia
Source: PLoS One. 2012 Jun 27;7(6):e39364. doi: 10.1371/journal.pone.0039364 (PMC3384666; doi:10.1371/journal.pone.0039364)
Supplement: Table S7 — Manual phalanx I-1 measurements. (DOC) [file pone.0039364.s007.doc]

Table S7: McI-1 measurements (mm)

|  | Left | Right |
| --- | --- | --- |
| Medial length | 104.05 | 101.73 |
| Lateral length | 108.93 | 102.6 |
| Longest length | 118.21 | 111.1 |
| Proximal width dorsal margin | 28.51 | 28.77 |
| Proximal width medial margin | 39.5 | 32.79 |
| Proximal width ventral margin | 33.52 | 37.62 |
| Proximal height | 33.97 | 31 |
| Distal width (dorsal) | 26.46 | 23.9 |
| Distal width (ventral) | 29.85 | 28.12 |
| Lateral condyle height | 33.61 | 33.19 |
| Medial condyle height | 30.62 | 29.18 |
| Mid-shaft width | 25.08 | 26.92 |
