# Supplementary material for: New Forearm Elements Discovered of Holotype Specimen Australovenator wintonensis from Winton, Queensland, Australia
Source: PLoS One. 2012 Jun 27;7(6):e39364. doi: 10.1371/journal.pone.0039364 (PMC3384666; doi:10.1371/journal.pone.0039364)
Supplement: Table S8 — Manual phalanx I-2 measurements. (DOC) [file pone.0039364.s008.doc]

Table S8: Right McI-2 measurements (mm)

| Proximal height | 59.84 |
| --- | --- |
| Proximal width | 26.08 |
| Articular facet height | 44.97 |
| Dorsal margin | 187.44 |
| Dorsal length | 150.34 |
| Ventral margin | 125.01 |
| Ventral length | 106.63 |
| Proximal height / width ratio | 2.29 |
