# Supplementary material for: New Forearm Elements Discovered of Holotype Specimen Australovenator wintonensis from Winton, Queensland, Australia
Source: PLoS One. 2012 Jun 27;7(6):e39364. doi: 10.1371/journal.pone.0039364 (PMC3384666; doi:10.1371/journal.pone.0039364)
Supplement: Table S9 — Metacarpal II measurements. (DOC) [file pone.0039364.s009.doc]

Table S9: Right Metacarpal 2 measurements (mm)

|  | Actual specimen | Estimated specimen length |
| --- | --- | --- |
| Medial length | 131.15 |  |
| Lateral length | 138.15 |  |
| Proximal width dorsal margin | 56.3 |  |
| Proximal width ventral margin | 33.14 |  |
| Greatest proximal width | 55.79 |  |
| Proximal height | 30.4 |  |
| Distal width (dorsal) |  | 28.99 |
| Distal width (ventral) |  | 37.86 |
| Lateral condyle height |  | 31.4 |
| Medial condyle height | 35.52 | 39.03 |
| Mid-shaft condyle height | 29.01 |  |
