# Supplementary material for: New Forearm Elements Discovered of Holotype Specimen Australovenator wintonensis from Winton, Queensland, Australia
Source: PLoS One. 2012 Jun 27;7(6):e39364. doi: 10.1371/journal.pone.0039364 (PMC3384666; doi:10.1371/journal.pone.0039364)
Supplement: Table S10 — Manual phalanx II-1 measurements. (DOC) [file pone.0039364.s010.doc]

Table S10: Right McII-1 measurements (mm)

| Medial length | 71.04 |
| --- | --- |
| Lateral length | 64.29 |
| Longest length | 84.9 |
| Proximal height | 40.9 |
| Proximal width | 31.86 |
| Distal width (dorsal) | 22.96 |
| Distal width (ventral) | 30.57 |
| Lateral condyle height | 29.41 |
| Medial condyle height | 27.38 |
| Mid-shaft width | 21.11 |
