# Supplementary material for: New Forearm Elements Discovered of Holotype Specimen Australovenator wintonensis from Winton, Queensland, Australia
Source: PLoS One. 2012 Jun 27;7(6):e39364. doi: 10.1371/journal.pone.0039364 (PMC3384666; doi:10.1371/journal.pone.0039364)
Supplement: Table S11 — Manual phalanx II-2 measurements. (DOC) [file pone.0039364.s011.doc]

Table S11: Left McII-2 measurements (mm)

| Medial length | 69.9 | 79.09 |
| --- | --- | --- |
| Lateral length | 71.34 | 80.69 |
| Longest length | 78.85 | 86.51 |
| Proximal height | 34.59 |  |
| Proximal width (ventral) | 26.43 | 34.77 |
| Distal width (dorsal) |  | 25.82 |
| Distal width (ventral) |  | 28.75 |
| Lateral condyle height |  | 29.57 |
| Medial condyle height | 31.12 |  |
| Mid-shaft width | 23.55 |  |
