# Supplementary material for: New Forearm Elements Discovered of Holotype Specimen Australovenator wintonensis from Winton, Queensland, Australia
Source: PLoS One. 2012 Jun 27;7(6):e39364. doi: 10.1371/journal.pone.0039364 (PMC3384666; doi:10.1371/journal.pone.0039364)
Supplement: Table S12 — Manual phalanx II-3 measurements. (DOC) [file pone.0039364.s012.doc]

Table S12: Right McII-3 measurements (mm)

| Proximal height | 55.13 |
| --- | --- |
| Proximal width | 24.88 |
| Articular facet height | 45.89 |
| Dorsal margin | 156.01 |
| Dorsal length | 137.66 |
| Ventral margin | 123.23 |
| Ventral length | 101.92 |
| Proximal height / width ratio | 2.21 |
