# Supplementary material for: New Forearm Elements Discovered of Holotype Specimen Australovenator wintonensis from Winton, Queensland, Australia
Source: PLoS One. 2012 Jun 27;7(6):e39364. doi: 10.1371/journal.pone.0039364 (PMC3384666; doi:10.1371/journal.pone.0039364)
Supplement: Table S13 — Manual phalanx III-1 measurements. (DOC) [file pone.0039364.s013.doc]

Table S13: Right McIII-1 measurements (mm)

| Medial length | 68.37 |
| --- | --- |
| Lateral length | 66.81 |
| Longest length | 73.11 |
| Proximal height | 27.57 |
| Proximal width (ventral) | 31.25 |
| Distal width (dorsal) | 14.04 |
| Distal width (ventral) | 24.31 |
| Lateral condyle height | 21.16 |
| Medial condyle height | 22.7 |
| Mid-shaft width | 20.74 |
