# Supplementary material for: New Forearm Elements Discovered of Holotype Specimen Australovenator wintonensis from Winton, Queensland, Australia
Source: PLoS One. 2012 Jun 27;7(6):e39364. doi: 10.1371/journal.pone.0039364 (PMC3384666; doi:10.1371/journal.pone.0039364)
Supplement: Table S14 — Manual phalanx III-3 measurements. (DOC) [file pone.0039364.s014.doc]

Table S14: McIII-3 measurements (mm)

|  | Left | Right |
| --- | --- | --- |
| Medial length | 37.56 | 38.98 |
| Lateral length | 36.28 | 37.72 |
| Longest length | 39.44 | 41.6 |
| Proximal height | 18.57 | 18.84 |
| Proximal width | 14.02 | 13 |
| Distal width (dorsal) | 18.26 | 18.09 |
| Distal width (ventral) | 13.74 | 13.98 |
| Lateral condyle height | 14.64 | 14.5 |
| Medial condyle height | 15.33 | 15.98 |
| Mid-shaft width | 11.44 | 10.84 |
