# Supplementary material for: New Forearm Elements Discovered of Holotype Specimen Australovenator wintonensis from Winton, Queensland, Australia
Source: PLoS One. 2012 Jun 27;7(6):e39364. doi: 10.1371/journal.pone.0039364 (PMC3384666; doi:10.1371/journal.pone.0039364)
Supplement: Table S15 — Manual phalanx III-4 measurements. (DOC) [file pone.0039364.s015.doc]

Table S15: Right McIII-4 measurements (mm)

| Proximal height | 31.07 |
| --- | --- |
| Proximal width | 12.87 |
| Dorsal margin | 79.98 |
| Dorsal length | 75.12 |
| Ventral margin | 70.31 |
| Ventral length | 62.14 |
