# Supplementary material for: New Forearm Elements Discovered of Holotype Specimen Australovenator wintonensis from Winton, Queensland, Australia
Source: PLoS One. 2012 Jun 27;7(6):e39364. doi: 10.1371/journal.pone.0039364 (PMC3384666; doi:10.1371/journal.pone.0039364)
Supplement: Table S16 — Neovenatorid measurements. (DOC) [file pone.0039364.s016.doc]

Table S16: Neovenatorid ratio measurements (mm)

| Femur length | 545.65 |
| --- | --- |
| Humerus length | 303.35 |
| Humerus / Femur length ratio | 0.56 |
| Deltopectorial Crest length | 116.88 |
| Humerus Deltopectorial Crest / Humerus length ratio | 0.38 |
| Deltopectorial Crest length inclusive of proximal end (Figure 2) | 143.13 |
| Humerus Deltopectorial Crest / Humerus length ratio inclusive of proximal end (Figure 2) | 0.47 |
